# Microstructural behavior of magnetorheological elastomer undergoing durability evaluation by stress relaxation

**DOI:** 10.1038/s41598-021-90484-0

**Published:** 2021-05-25

**Authors:** Mohd Aidy Faizal Johari, Saiful Amri Mazlan, Mohamed Mahmoud Nasef, U. Ubaidillah, Nur Azmah Nordin, Siti Aishah Abdul Aziz, Norhasnidawani Johari, Nurhazimah Nazmi

**Affiliations:** 1 Engineering Materials and Structures (eMast) ikhoza, Malaysia-Japan, International Institute of Technology (MJIIT), Universiti Teknologi Malaysia, 54100 Kuala Lumpur, Malaysia; 2 Advanced Materials Research Group, Centre of Hydrogen Energy, Institute of Future Energy, Universiti Teknologi Malaysia, 54100 Kuala Lumpur, Malaysia; 3 Mechanical Engineering Department, Faculty of Engineering, Universitas Sebelas Maret, J1. Ir. Sutami 36A, Ketingan, Surakarta, 57126 Central Java Indonesia

**Keywords:** Engineering, Materials science, Physics

## Abstract

The widespread use of magnetorheological elastomer (MRE) materials in various applications has yet to be limited due to the fact that there are substantial deficiencies in current experimental and theoretical research on its microstructural durability behavior. In this study, MRE composed of silicon rubber (SR) and 70 wt% of micron-sized carbonyl iron particles (CIP) was prepared and subjected to stress relaxation evaluation by torsional shear load. The microstructure and particle distribution of the obtained MRE was evaluated by a field emission scanning electron microscopy (FESEM). The influence of constant low strain at 0.01% is the continuing concern within the linear viscoelastic (LVE) region of MRE. Stress relaxation plays a significant role in the life cycle of MRE and revealed that storage modulus was reduced by 8.7%, normal force has weakened by 27%, and stress performance was reduced by 6.88% along approximately 84,000 s test duration time. This time scale was the longest ever reported being undertaken in the MRE stress relaxation study. Novel micro-mechanisms that responsible for the depleted performance of MRE was obtained by microstructurally observation using FESEM and in-phase mode of atomic force microscope (AFM). Attempts have been made to correlate strain localization produced by stress relaxation, with molecular deformation in MRE amorphous matrix. Exceptional attention was focused on the development of molecular slippage, disentanglement, microplasticity, microphase separation, and shear bands. The relation between these microstructural phenomena and the viscoelastic properties of MRE was diffusely defined and discussed. The presented MRE is homogeneous with uniform distribution of CIP. The most significant recent developments of systematic correlation between the effects of microstructural deformation and durability performance of MRE under stress relaxation has been observed and evaluated.

## Introduction

The interest in the development of magnetorheological elastomers is fast growing due to their ability to respond and behave relative to the external magnetic stimuli. This surpasses the potential of MRE beyond conventional materials and acknowledged scientifically as smart materials. Distinctively, magnetorheological elastomer (MRE) is composed of a soft elastomeric matrix that is embedded with magnetizable particles at the desired proportion. Possess a viscoelastic performance, MRE stiffness and damping behavior can be rapidly, continuously, and reversibly controlled by an external magnetic field^1–3^. These changeable properties bring MRE at the highest degree of hope to be used in prominent adaptive engineering applications as currently available for restrictedly small deformation and vibration control^4–6^. Existing research^7^ recognizes the critical role played by MRE on a par with similar materials such as rubber and elastomer. MRE has increasingly reached into new engineering applications and bound with irrefutable scientific procedure evaluation, particularly on its long-term performance ostensibly durability.

Durability however has long been a question of great interest in a wide range of engineering material fields and plays a fundamental role in determining material resistance to any change in property levels due to the service environment^8^. The durability of MRE and its consequences are an important, but understudied, cause for concern. Recent years have witnessed a growing academic interest in MRE durability however, the research to date, has not been able to convincingly show that specified criteria available for the MRE durability test^9^. At present, the majority of MRE durability studies were focused on the load at tensile and compressive modes^10,11^. The work of Wang et al.^10^ for example, applied the with cyclic tensile stress at a maximum displacement of 25, 50, 75 and 100 times its original length and cyclic loaded at 300 rad/min test frequency. Another research^11^ conducted fatigue test on natural rubber MRE with strain applied over a sample length of 70 mm for up to 500 cycles. Correspondingly, what we know about the testing mode is mostly based upon laboratory studies that investigate how durability performance effected by the MRE sample^9^. Having said that, for the viscoelastic behavior of MRE, other explorations on different modes of failure load such as torsional, oscillating, and sliding are getting good prospects and may also be further investigated. In addition, the recent review article^12^ reported the significance of a repeat-cycle investigation involving the MRE modulus. Experimental works conducted so far were implemented shearing mode in purpose to be more realistic and reliable condition related to interaction within particles and matrix molecular structure chain^13^.

Stress relaxation study on potential smart materials^14–16^ has led to a proliferation of variables that have been found to affect the properties of the material. However, it has been conclusively shown that, no data on the extensive connection between stress relaxation and microstructural behavior of MRE undergone durability were found in the literature review, apart from the recent preliminary research^17^. Most of the previous studies evaluating stress relaxation observed results in whether mathematical modeling, mechanical or rheological characteristics. Stress relaxation has been utilized for the time-dependent viscoelastic properties of polymer materials^18–23^. There were few investigations have concentrated on the stress relaxation of MRE reported to date^24,25^. The influences of constant strain level, matrix, magnetic field, and temperature on the stress relaxation behavior of MRE was hitherto investigated^24^. The results show that stress relaxation directly depending on these parameters by restricting the slippage and stretching of the molecular chain. The study conducted^25^ was mainly focused on the modeling of stress relaxation behavior. Likewise, there was no investigation made on the morphological study which is believed to have a strong correlation to the change in resultant properties of MRE. Although the studies have established dependency, the study has yet to examine the associated microstructural effects of stress relaxation in a more systematic manner. There is a current paucity evidence-based literature relating to this phenomenon.

A comprehensive study on microstructure behavior under stress relaxation is very indispensable to understand how this phenomenon influences the property of MRE. Most of the microstructure studies on MRE were related to the justification of pre-structural^26–28^ and particle arrangement^2,29–31^ in the matrix. Investigation^32^ found that the interaction mechanism leads to the links with the shear property, which necessary for the design of MRE based devices. However, the mechanisms that underpin MRE microstructural behavior are not fully understood. The work in^23^ explained the utmost fascinating phenomena that occurred to the sheared amorphous system is strain localization. Again, the extent to which stress and strain influence on the microstructure of MRE remains unknown. At the elastic rubbery region of viscoelastic MRE, entanglements, or cross-linkages of the amorphous structure plays a main role in microstructural behavior and validated with the time–temperature condition^18^. In general, the determination of MRE overall microscopic behavior was treated as an integrated evidence to the approach of theory and experiments. Some of the research conducted on MRE were discussed on the microstructure effect in general and related to the modeling approach^21,33,34^. Otherwise, few compelling works^18,20,21,35,36^ on viscoelastic polymeric materials were implemented to the MRE evaluation in this study.

Limited studies reported on the use of an Atomic Force Microscope (AFM) has provided additional capabilities and advantages emulate to other microscopies instrumentation. However, it is unfortunate that the utilization of AFM in MRE studies is scarce as indicated by a few investigations reported in literature^20,37–40^. To date and the best of authors’ knowledge, there is no comprehensive studies have been published on the utilization of AFM for the verification of deformation by stress relaxation and strain localization at the microscopic level. Hence, AFM execution in investigating these stress relaxation and strain localization phenomena in MRE will be the technical originality of this work. Following the literature, the corresponding summary of the major contribution of this present study can be summarized in Table 1.

**Table 1:** Comparison between the contribution of the present study towards understanding of microstructural behavior of MRE subjected to stress relaxation and previous studies.

Materials	Investigated elastomeric and amorphous behavior	Test mode	Stress relaxation evaluation	Strain (%)	Duration (s)	SEM evaluation		AFM evaluation		Major contributions and findings	References
						Before	After	Before	After		
Polymer	Yes	Modeling	Yes	-	-	No	No	No	No	The study introduced a model for amorphous structure relaxation	^23^
Hydrocarbon rubber and polymers	Yes	Modeling	Yes	-	-	No	No	No	No	Discussed the relation between the structure of polymeric materials and their viscoelasticity For the assessment, the analysis presented a mathematical model	^18^
Natural rubber and carbon black	Yes	Uniaxial tensile	Yes	100–600	25,200	No	No	No	No	Introduced model and discover relaxation behavior at high strain	^19^
Polyurethane and nanoclay	Yes	Uniaxial tensile and Modeling	Yes	10–200	2400	No	No	No	No	Characterized stress relaxation and creep related to microphase separation	^20^
styrene-butadiene rubber and silica	Yes	Torsional Shear	Yes	0.1	25,200	No	Yes	No	No	Investigated morphology and rheological evolution influenced by nanoparticles	^21^
Rubber-modified polystyrene	Yes	Flexural	Yes	0.53–2.45	30	No	No	No	No	Measured material resistance at short-time stress relaxation	^22^
Polyurethane/epoxy CIP 70 wt%	Yes	Torsional Shear	Yes	1–10	1000	No	No	No	No	Indicated influenced by particle content, magnetic field, and temperature to the stress relaxation. Introduced theoretical predictions of stress relaxation	^24^
polyurethane/epoxy CIP 60 wt%	Yes	Torsional Shear and modeling	Yes	0.5–2	1000	No	No	No	No	Application of viscoelastic fractional derivative model to the stress relaxation modulus	^25^
Natural rubber nanocomposite	Yes	Uniaxial tensile and Modeling	Yes	50	5400	Yes	No	No	No	Measured effects of blend composition, filler polarity, and temperature on stress relaxation. The study employed theoretical models and fitted with an experimental curve	^36^
Polydimethylsiloxane CIP 75 wt%	Yes	Phase-contrast AFM	No	No	No	Yes	No	Yes	No	Determination of shape and size distribution in magnetorheological composite by atomic force microscopy	^37^
Butadiene—styrene rubber and schungit filler 39 wt%	Yes	Uniaxial tensile and tapping mode AFM	No	330	No	No	No	Yes	No	Comprehensive study on physical–mechanical data by atomic force microscopy	^38^
Silicone rubber and CIP 20–60 wt%	Yes	Non-contact AFM	No	No	No	No	No	Yes	No	Investigated microstructure, surface magnetic and elastic properties of MRE by nanoindentation method atomic force microscopy	^39^
Polybutadiene polyol-based polymer and CIP 50–70 wt%	Yes	DMA and tapping mode AFM	No	0–3	No	Yes	No	Yes	No	Identification of significant change in the structure of MRE matrices using atomic force microscopy featuring soft and hard segments	^40^
Silicone rubber and CIP 70 wt%	Yes	Torsional Shear and tapping mode AFM	Yes	0.01	84,000	Yes	Yes	No	Yes	The present study conducted a stress relaxation test by torsional shear at the lowest ever reported strain % and longest duration ever investigated for MRE. Microstructure behavior of before and after durability was observed by scanning electron microscope and proposed the failure mechanism. This present study also introduced the phenomena of molecular slippage, disentanglement, microplasticity, microphase separation, and shear bands to the MRE amorphous matrices molecular system. Confirmation of soft and hard domain after durability by stress relaxation was evaluated using an atomic force microscope	Present study

The present study provides additional evidence to the current literature and has gone some way towards enhancing our understanding of microstructural behavior subjected to stress relaxation. Further continuous investigation of MRE is necessary to improve the properties and to understand the behavior towards the different testing and characterization procedures^12^. Therefore, it is always worthy to further investigate the shear mode deformation and long-term behavior of MRE. It will therefore be of the utmost importance to consider microstructural conditions along with durability performance. In this work, characteristics of the MRE sample suffered from the stress relaxation durability evaluation test and the degradation mechanisms were evaluated through Field Emission SEM (FESEM) and Atomic Force Microscope (AFM).

The aim of this work is to study the microstructural behavior of MRE undergoing durability by stress relaxation. All aspects contributed to the long-term durability behavior of MRE brings the opportunity to be discussed. However, this present study is being implemented as an initial step towards the sharing of new information in the field and, to date, few contributing parameters, such as the various particle fraction and distribution, may not yet be considered. For this purpose, this paper is organized as follow. Consecutive to the introduction, results and discussion presents the details characterization of stress relaxation and microstructural analysis of the MRE sample. The main finding will be summarized in the same results and discussion section. The preparation of MRE and methodology for stress relaxation measurement is described in the method section. The methodology for morphological properties evaluation by FESEM and AFM is also reported in the same section.

## Results and discussion

### Stress relaxation

**Figure 1:**
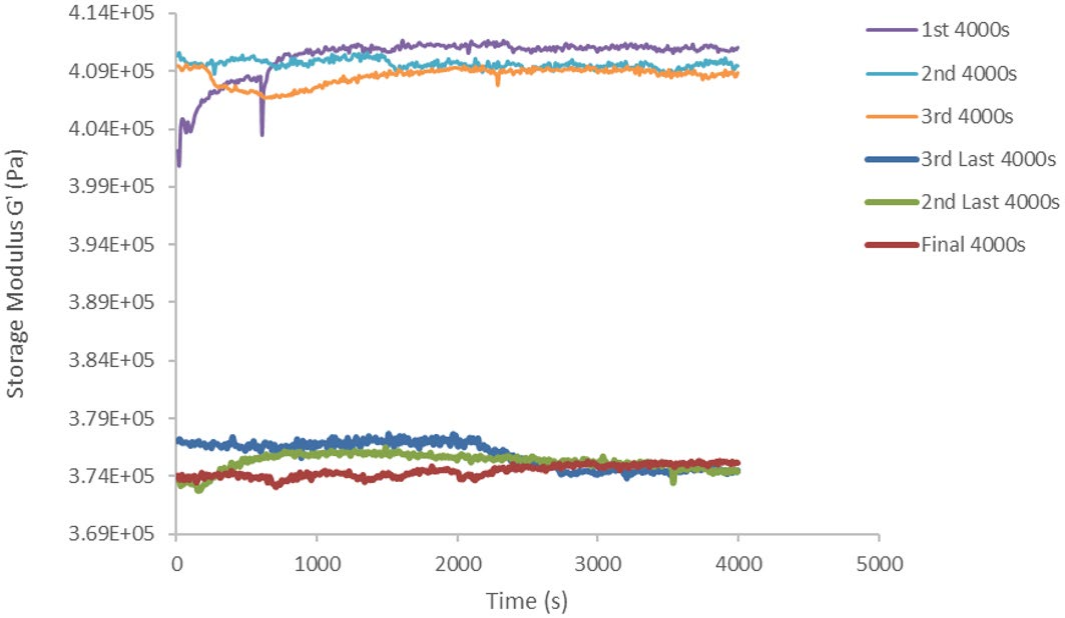
Performance reduction of 8.7% storage modulus corresponding to test time of initial and final phase intervals at a constant 0.01% strain for total test duration of 84,000 s.

The performance of the MRE sample was first evaluated by its ability to store deformation energy elastically through storage modulus characterization. The plotted graph in Fig. 1 shows the comparability assessment of this behavior corresponds to the early stage durability in the range of 0–12,000 s, which indicates as 1st, 2nd and 3rd interval in the graph, and the final range from 72,000 to 84,000 s, at indication of 3rd last, 2nd last and final interval in the graph’s legend. There were three set of 4000 s intervals for each range. As can be seen, the characteristic graph features distinctly show a storage modulus lowering trends with the increase in the test time at a constant 0.01% of strain. At the initial phase, storage modulus values were steadily decreased after the first 4000 s but with less diminution reaching to the third interval of test time. This behavior may be due to the MRE system that has microstructurally reversible aligns at still higher modulus of resilience.

However, at instantaneous stress continuously applied to the system, the molecular chains were stretched, disentangled, and more expeditiously accumulated with the cross-linked of the MRE matrix. This continuous applied stress at similar constant strain has leisurely decreased the proportionality behavior of the MRE, and the covalent cross-linkages compromising the return-ability of the stretched chains. However, at a wider range of test duration up to 84,000 s, localized strain and microplasticity were suspected to be developed in the system. Furthermore, at a larger scale localization associated with the test time increase, the development of plasticity at the molecular level within the narrow regions of shear bands has imparted softening behavior to the MRE. As a result, the storage modulus was observed to decrease by 8.7% at the end of the test duration. Nevertheless, the storage modulus in the final phase interval performed slightly different from the beginning phase interval. Throughout the process of cumulatively decreased in storage modulus, the elastic response of MRE energy-storing ability has reached a plateau, attributed to the stability of the molecular structure of the MRE.

**Figure 2:**
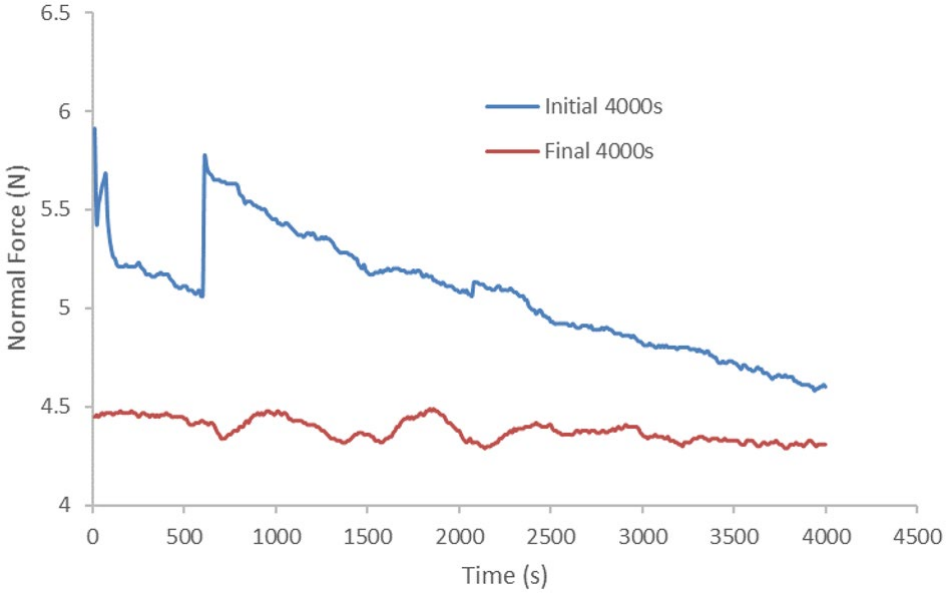
Normal force of MRE under durability shear test at total test duration of 84,000 s.

Throughout the test, the MRE sample notably suffered from the shear-contact force generated from the rheometer apparatus by a parallel rotary plate. This is associated with the shear property by the interaction between particles and the interactions between the particles and the matrix, which is similar to the mechanism associated with the normal force results^32^. Figure 2 shows the experimental results of normal force under oscillatory shear test. The sample was pre-compressed with the contact force to avoid slippage between the sample and the shear plate during the test. The pre-compression produced an initial 5.9 N normal force on the sample while the compressive strain was maintained constant. In this work, the interesting circumstances are the shear-deformation durability related to normal force throughout 4000 s. A steeper lessening trend was observed for the normal force at the beginning of 4000 s interval of the durability test, with some distortion and micro-changes while reaching the earliest 600 s of the shear oscillating time. The data accumulation at this point has just reached stability of the sample and this phenomenon was suspected to be associated with the random arrangement of the molecular chain structure, which suddenly slid between particles-matrix and the presence of micro-sized voids.

Continuous evaluation led the sample to be more stable and consistent in managing molecular restructuring and captivating with applied normal force and stress. However, the constant strain of 0.01% executed throughout the test thus resulting in the development of larger localized failure. The MRE sample was strained up to 84,000 s and the additional continuous stress applied to the sample was then distributed to the localized region of the molecular chain structure. As the sample held for constant deformation, the stress in the sample was reduced with time and this phenomenon is likely to be responsible for stress relaxation. The shear plasticity is assumed to occur through a series of this molecular local restructuring. Besides of fluctuated micro-stress at the molecular level as discussed earlier, the MRE sample was suffered from microplasticity, where the sample was all-encompassing in the elastic domain while some local areas were in the plastic domain. This permanent transformation has somehow reduced the elastic potentiality of the MRE system. Eventually, normal force was gradually reduced throughout the test associated with this micro-plasticity deformation by approximately 27%. Undisputable observation finds that normal force was solely reduced expeditiously during the initial phase and lessen imperceptibly as reached to the final phase. Thus, an assumption can be made that with longer duration, the sample microscopically attains the stability and therefore, lesser effort for deformation would take place. Consequently, the incremental value of normal force applied on the sample while sheared, has been found to be indiscernible along the process.

**Figure 3:**
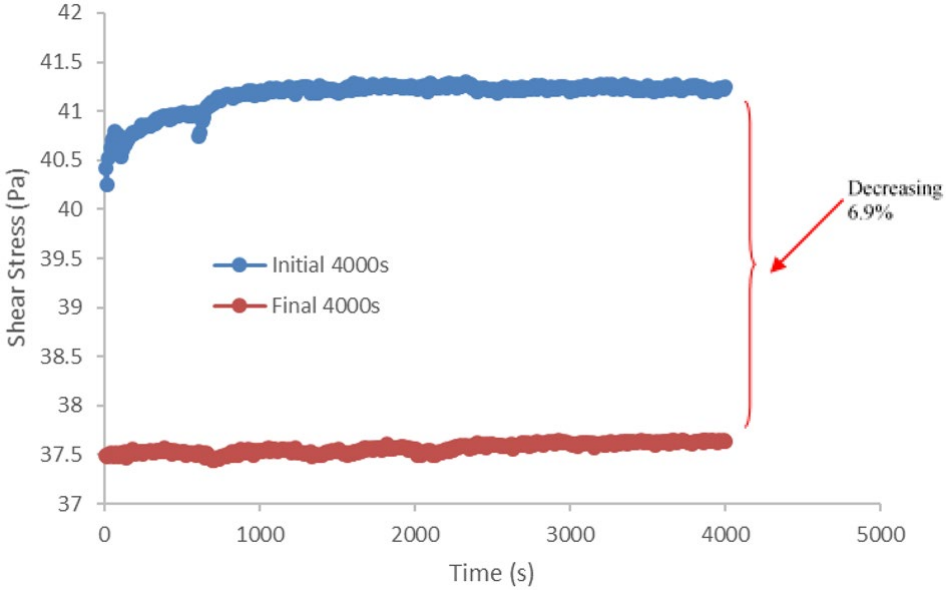
Stress relaxation stress-time curve for MRE under durability shear test for 84,000 s test duration.

The long-term behavior prediction from time-dependent viscoelastic properties of MRE was investigated by stress relaxation. In the course of stress relaxation, stress deteriorated with time under conditions of constant deformation as shown in Fig. 3. In this study, the sample was tested up to 84,000 s and constant strain at 0.01%. At the beginning of the 4000 s time interval, the curve shows an initial instantaneous stress which increased with the increase in relaxation time. A slight increase in the stress relaxation rate, which then endured with balanced stress as indicates from the graph was agreed with the fact that the molecular chain of the matrix MRE was stretched, slide, and aligned according to the stress applied simultaneously along the process. At tremendously low strain levels, the sample took less time to achieve the balance stress. However, the interminable shear stress that has been applied and rapidly strained at a fixed deformation led to the structure relaxation mechanisms such as molecular relaxation, disentanglement of cross-links, and structural rearrangement. These mechanisms were contributed to the degradation of the sample stress relaxation’s resistance property. As a result, by the end of durability evaluation, the stress relaxation behavior was decreased by approximately 6.9% with the increase of shear durability test time, particularly between the initial and the final phases of the test duration. This trend has particularly met the rubbery plateau characteristic of viscoelastic materials and agrees with literature^18^.

In this study, the durability was subjected to the stress relaxation, in which the sample was kept sheared with a constant strain throughout the entire test duration as an additional deformation beyond the assigned strain limit was impossible to have occurred. Therefore, stress relaxation can only occur on the molecular level. The relaxation mechanism that occurred in the sample was observed microscopically to prove that the decrease in the relaxation behavior was related to the disentanglement of cross-links and structural rearrangement of the molecular chains, which may occur from molecular rupturing. That behavior is common in the amorphous polymers, pertinently to the sample used in this study. Additionally, details of the microscopic investigation of this original durability evaluation method has led to interesting and novel findings related to this phenomenon.

### Microstructure characterization

**Figure 4:**
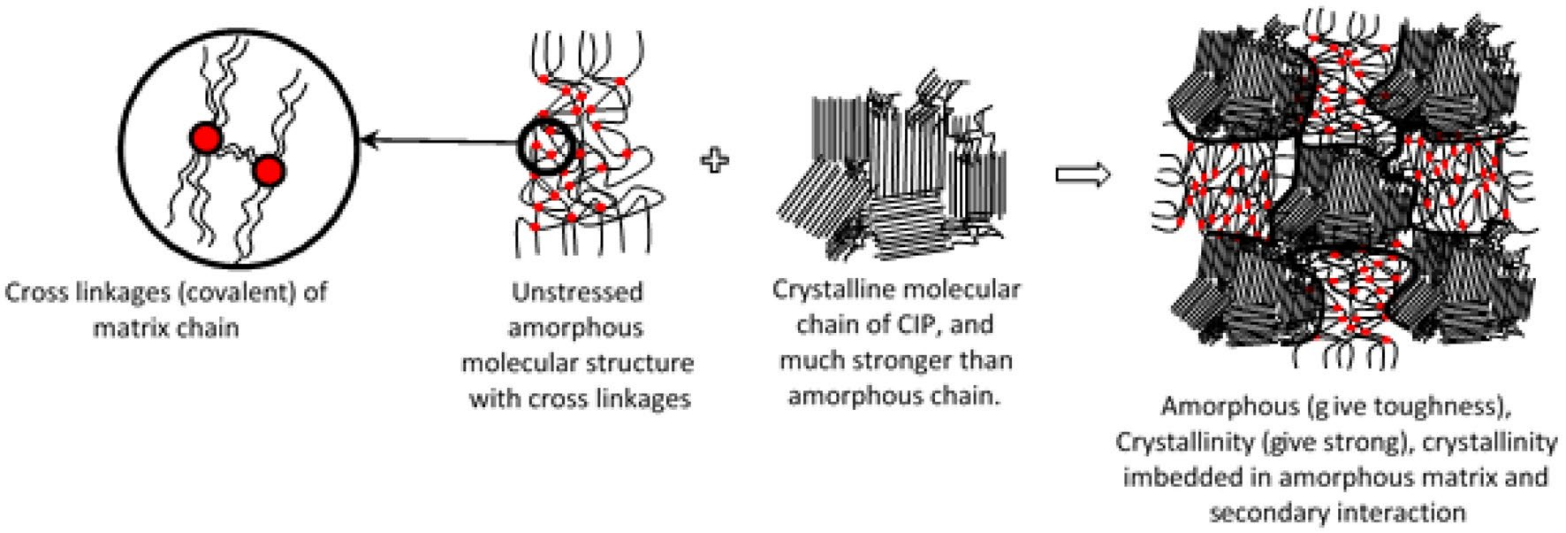
Schematic illustration of cross-linkages, molecular chain structure for matrix, particle, and cured MRE.

The theoretical concept of molecular chain structure and cross-linkages in sheared amorphous solid was ideally relevant to MRE evaluation due to its matrix amorphous molecular structure^18,23,41^. Inaugurate to the conceptualization for MRE, molecular chain structure and cross-linkages of cured MRE can be simplified into schematic details as in Fig. 4. Cross-linkages developed during the curing process of MRE is determining its elasticity and deformation limits. Such cross-linkages represent a critical structural factor that imparts high elastic properties and made the MRE system more rigid to prevent any viscous dominant response.

**Figure 5:**
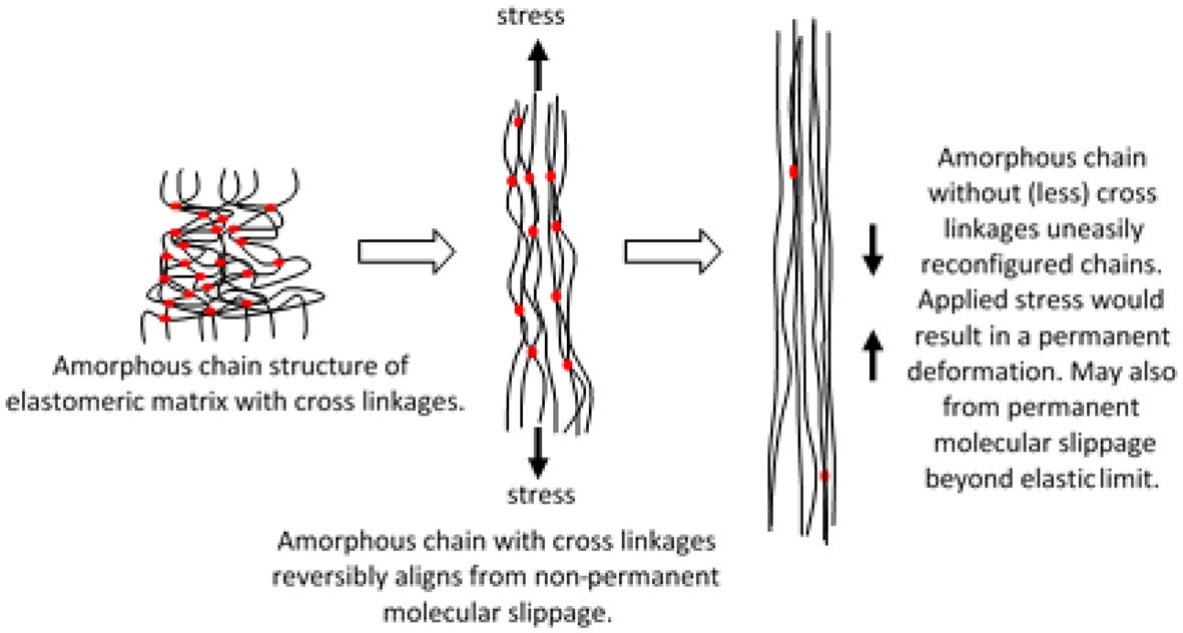
Schematic representation of matrix amorphous molecular chain stretched at permanent and non-permanent deformation.

Stress relaxation happened on the molecular scale and involved changing the molecular chain arrangement. Besides, phenomena of stress relaxation occurred through a variety of mechanisms including disentanglement of cross-links, elastic stretching, inelastic deformation, structural change by phase transformation, structural rearrangement due to rupturing, microphase separation, microplasticity and shear bands formation by localized strain. The fundamental theoretical concept of molecular deformation of amorphous chain structure is illustrated in Fig. 5. As the MRE was imposed by the incessant shear stress at constant strain, the stress was distributed over the molecular structure and this would disentangle the chains to create the molecular slippage. As more slippages and losses of cross-links occur in the MRE, the molecular structure becomes inadequately able to be reconfigured and resulted in a permanent deformation, as shown in the schematic figure.

**Figure 6:**
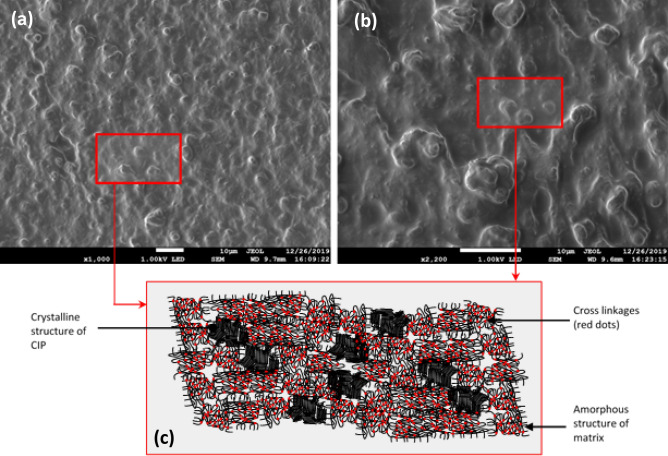
FESEM images of particle dispersion in isotropic distribution at: (**a**) low magnification and (**b**) high magnification of MRE before durability shear test. A schematic representation of both, crystalline structure containing CIP and amorphous matrix molecular structure (**c**).

MRE sample with 70 wt% CIP and 30 wt% silicon matrix is cured without the magnetic field and homogeneous distribution of the particles was observed with some agglomeration of particles as shown in FESEM images in Fig. 6a,b. As can be seen from the image (a) and its magnified version, the protruding granulates are CIP and the background base material is the silicon rubber. Before the durability evaluation, CIP seems uniformly distributed over the silicone rubber and it varies in sizes conforming to the description given by the supplier as stated in the experimental details (3.8–5.3 μm). A schematic representation of the sample with randomly dispersed particles (isotropic) manifesting how rigid spheres laid in the un-deformed state is shown in Fig. 6c. Before the durability test, amorphous molecular structures of the matrix were un-stretched with initial cross-linkages position generated after cured with the curing agent. The CIP is represented by a rigid sphere of crystalline molecular structure arranged randomly within the matrix amorphous molecular structure.

Having an amorphous molecular structure, the sheared samples at this low strain experienced an interesting phenomenon, which is called strain localization in which the materials deformed plastically in very narrow regions referred to as shear bands^23^. Even though this phenomenon occurs typically at very low strain, the shear velocity gradient during the test has dominantly affected the development of the shear bands comprising their size and shapes. The velocity gradient develops across the sample during shear is higher at the most outer section and diminishes towards the center of the sample. In this study, the shear profile generated across the sample was found very much affected by the shear band development and its mechanism.

**Figure 7:**
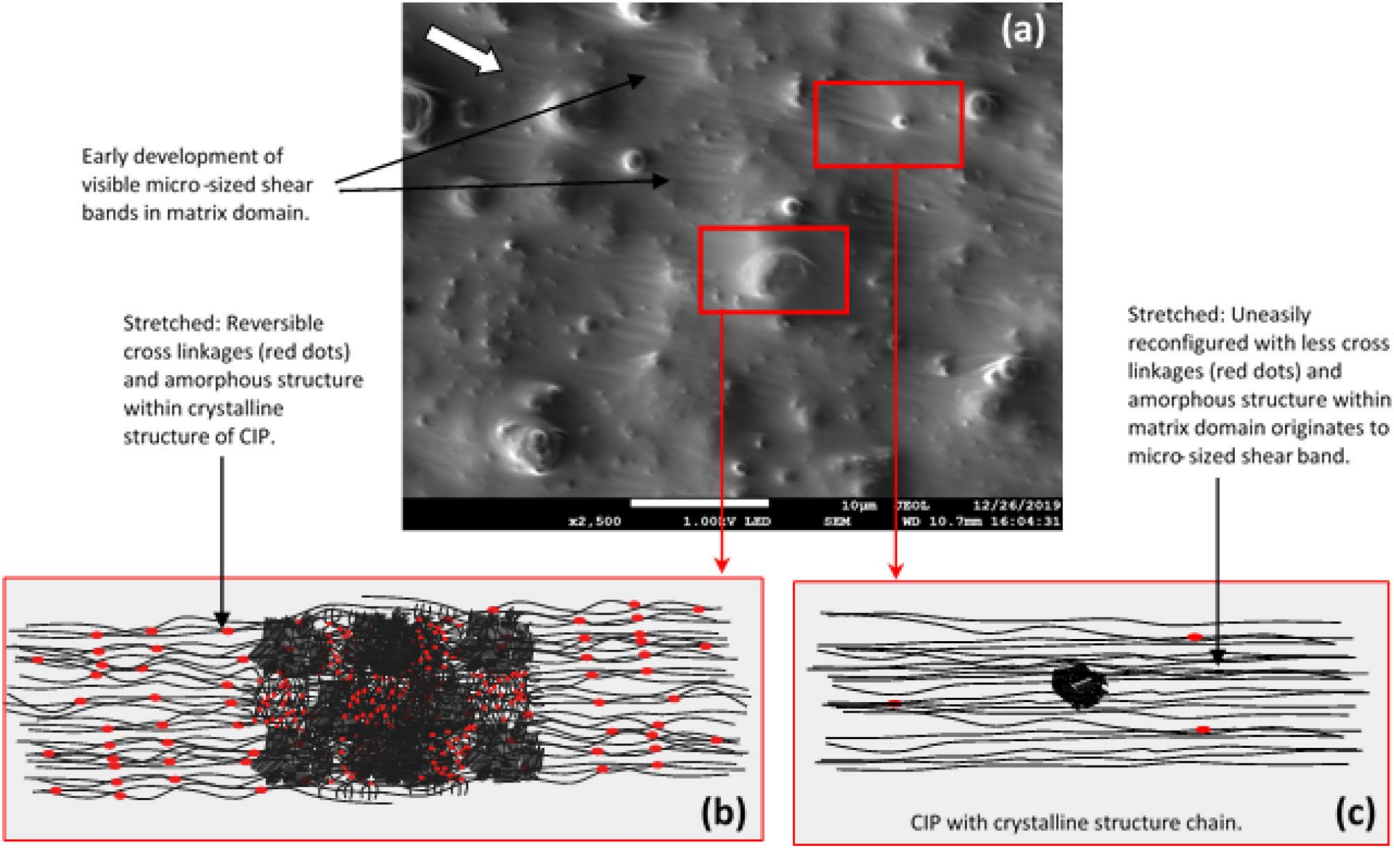
FESEM image of shear deformation at: (**a**) closer to the center of the sample (the white arrow represents the direction of shear stress applied) (**b**) schematic representations of molecular structure within the crystallographic plane, and (**c**) schematic representations of the stretched and broken cross-linkages of molecular structure.

Figure 7a shows the FESEM image of shear deformation closer to the center of the sample after durability evaluation (84,000 s). The FESEM image is generated from Piece 4 as shown in Fig. 15. As the sample was focused closer to the center region, the shear band was almost invisible, and apparent tiny stretched mark was uniformly developed as shown in the figure. Such tiny marks are due to the lesser stress experienced by the region, attributed to the different velocity gradient of shear stress across the sample. Besides, indiscernible marks around the particle and development of minuscule patterns on the matrix, it can be schematically represented as shown in Fig. 7b,c. Figure 7b demonstrates the bond stretching along the crystallographic plane and amorphous structure stretched with some non-permanent molecular slippage.

However, the stretched region was just below the localized elastic limits among the entangled amorphous molecular chains, and cross-linkages were then reversibly aligned. No permanent deformation occurred during the stretching at this stage, therefore no visible marks can be noticed. In contrast within the matrix domain, some cross-linkages of the molecular structure were uneasily reconfigured and less survived by breaking the cross-linkages from the stretching process. The disentanglement of the molecular structure has resulted in the slippage and breaking of the interconnected cross-linkages especially beyond the elastic limit of the localized phase. Steady shear stress that applied through the test has brought this phenomenon to the permanent deformation and forced it to remain localized in the confine shear band, as observed in Fig. 7c.

**Figure 8:**
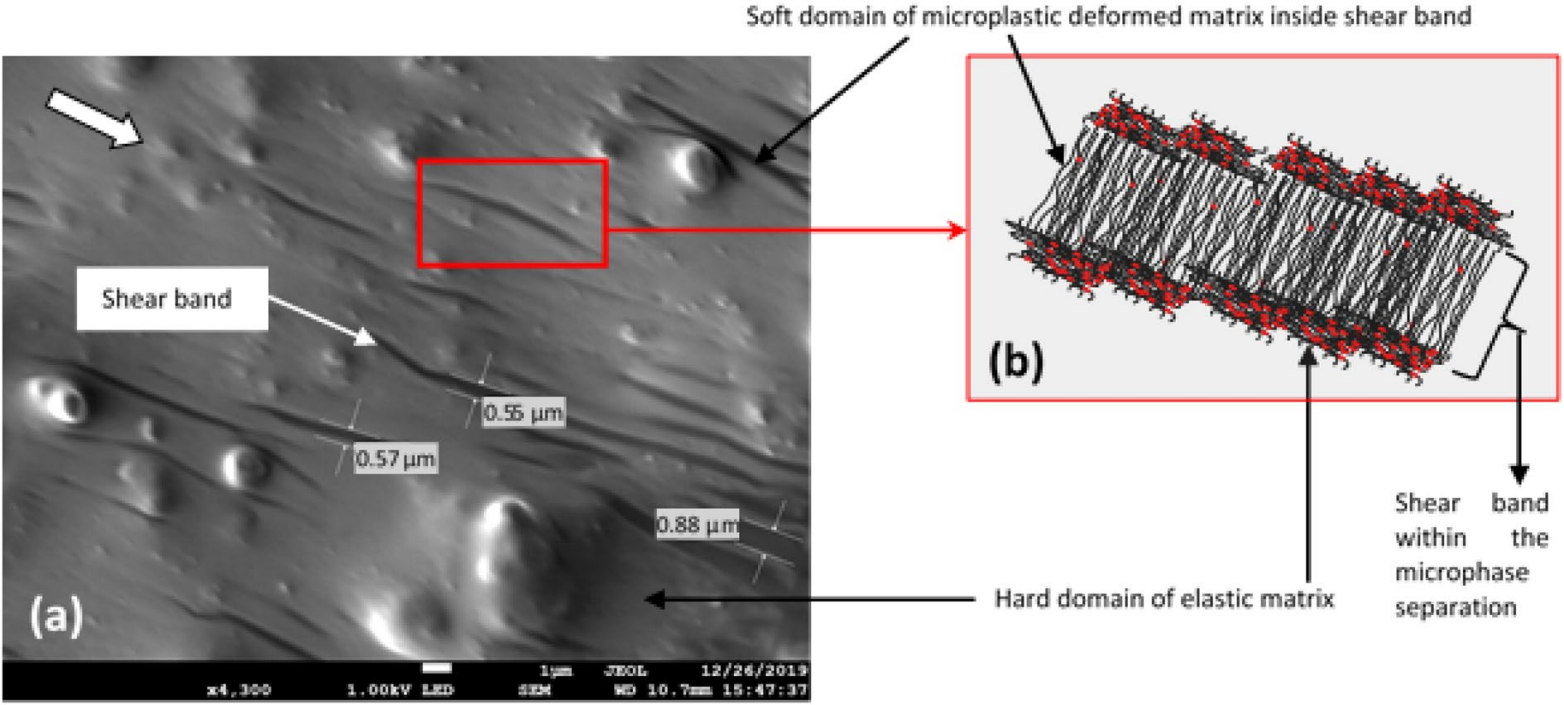
FESEM image of microscopic shear band (**a**) shear bands formation (the arrow represents the direction of shear stress applied) (**b**) schematic representations of stretched molecular structure and microphase separation.

Moving further from the center region, shear bands that have been developed by strain localization are more visible as shown in the FESEM image in Fig. 8a. The FESEM images taken from Piece 1, 2 and 3, as in Fig. 15 produce comparable patterns of shear bands. Three samples to verify the phenomenon of the shear band offered adequate evidence for to summarize the characteristics of the mechanism throughout this discussion. More permanent molecular slippage occurred, and cross-linkages of amorphous chains were broken as a consequence of uneasily reconfiguration and this phenomenon has simultaneously softened the matrix chains. Splitting the soften chains and abided harder domains of the elastic matrix have produced microphase separation, between the elastic and microplasticity deformations.

Microphase is the representative of the micro-sized domain of the matrix whereas the shear band falls within this phase separation. At this stage, a microscopically shear band was identified having an average thickness of < 1 µm (0.5–0.8 µm). The stretched chain in this phenomenon was destroyed and diminished the covalent bond of the cross-linkages as illustrated in Fig. 8b. The continual shear load has repeatedly softened the chains causing their pending to be exceeding the elastic limit for this localized region. Subsequently, the plastic flow has likely taken place in the inner portion of the molecular chain, slightly beyond the localized limit. However, there must be an outer region belong to the elastic matrix domain, which is still elastically strained, because the stress in this domain is falling below the elastic limit^42^.

On the other hand, towards the edge of the sample, shear stress was completely contributed to the angular displacement of the sample. This region having a maximum shear velocity gradient and stress relaxation through molecular motion. The motion was produced from the stretched molecular chain of the amorphous matrix domain. As the stretching magnitude is kept constant, no macroscopic movement is possible. Therefore, stress relaxation of the molecular structure has been mainly subjected to the disentanglement of the amorphous molecular chains in the soft domain of the matrix, at the micro-level. Furthermore, fluctuating stress developed in the amorphous molecular chain is attributed to the deformation and the rupture of the chain together with the cross-linked at soft dominated domain^20^. The diversity of the soft domain chain structure was contributed by the homogenous distribution of CIP, which correlated to the reduction of the elastic matrix region.

**Figure 9:**
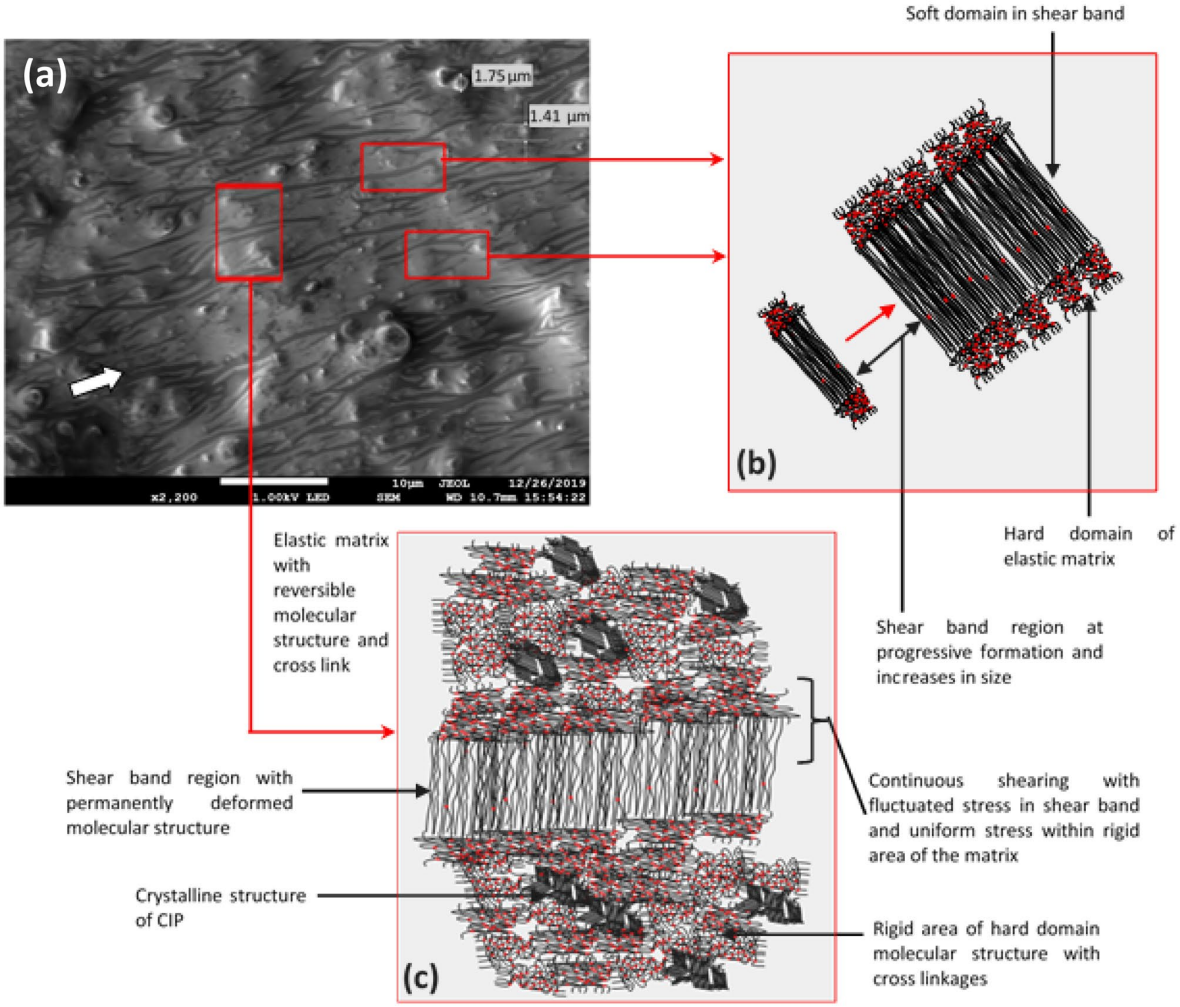
FESEM image of stress relaxation characteristic (**a**) sheared MRE after durability test (the arrow represents the direction of shear stress applied) (**b**) schematic representations of progression shear bands development (**c**) schematic representations of microphase separation within rigid and shear bands (soft) region.

As presented in Fig. 9a, the FESEM image shows the uniformed formation of shear bands visibly as stretched marks of microplasticity. In general, a clear formation of localized shear bands was achieved after the system is optimally sheared. The strain localization appears not to be persistent in this region and contributed to the uncertainty form of plastic strain. The plastic straining of the amorphous rubbery matrix at the molecular chain level has turned into stretched marks within the matrix, in which this phenomenon was not observed in the original sample before the test (Fig. 6).

As the shear velocity gradient is greater within this area, the shear bands become progressively thicker. The deformation mechanism moves through the shear direction and the deformations remain localized along the period of test duration. On average, the shear band deformation corresponded to the molecular chain disentanglement, breaking of cross-linkages, and the sequence of molecular slip events that contributed shear bands to become homogenous. Figure 9b illustrates the schematic progression of the localized matrix amorphous structure towards stress fluctuation developed by continuous shearing. Due to stress relaxation, the remaining rigid region however acquired rather a uniform distribution of stresses, while large fluctuations remained within the shear bands^23^ as demonstrated in Fig. 9c. At a longer period of test time, a range of elastic interaction, slip and rupturing of molecular chains, spatial distribution, and localization of strain have become apparent with the sighting of the shear bands and microplasticity marks, which obliquely aged the sample associated with the time elapsed.

**Figure 10:**
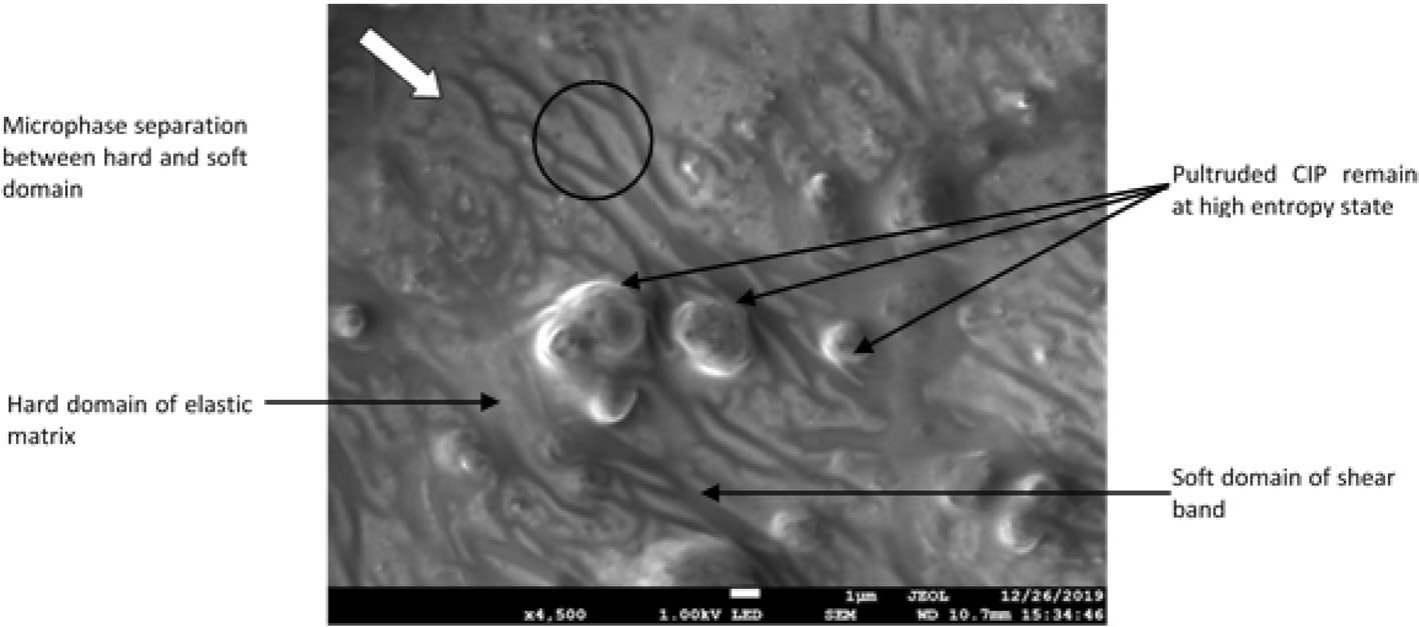
FESEM image of particles and matrix condition of MRE after durability test (the arrow represents the direction of shear stress applied).

The condition of CIP and silicon matrix after durability evaluation, particularly after 84,000 s test duration can be closely observed in Fig. 10. At several locations, particles demonstrated more protruding out from the localized area surrounded by the shear bands deformation. This may denote a notable characteristic of the condition of long-term stress relaxation. This fascinating phenomenon may have led to the embracing of the dispersed CIP and some portion of the enclosed matrix between them, molecularly known as secondary interaction. Secondary interaction in this study is referred to as the limited restrained region between matrix amorphous and CIP crystalline molecular chain structure. Secondary interaction was developed by a crystallinity molecular structure embedded in the amorphous matrix and this interaction is much stronger than the amorphous structure itself due to the presence of CIP. In the amorphous matrix, the uniformed dispersal of particles has increased the diversity of soft localized regions and simultaneously promoted the microplasticity deformation of shear bands through the entire sample area. The potential of hard domain in this soft localized region decreased and the microphase separation between hard and soft domains imparted by shearing shoved away the particles to remain at a high entropy state.

**Figure 11:**
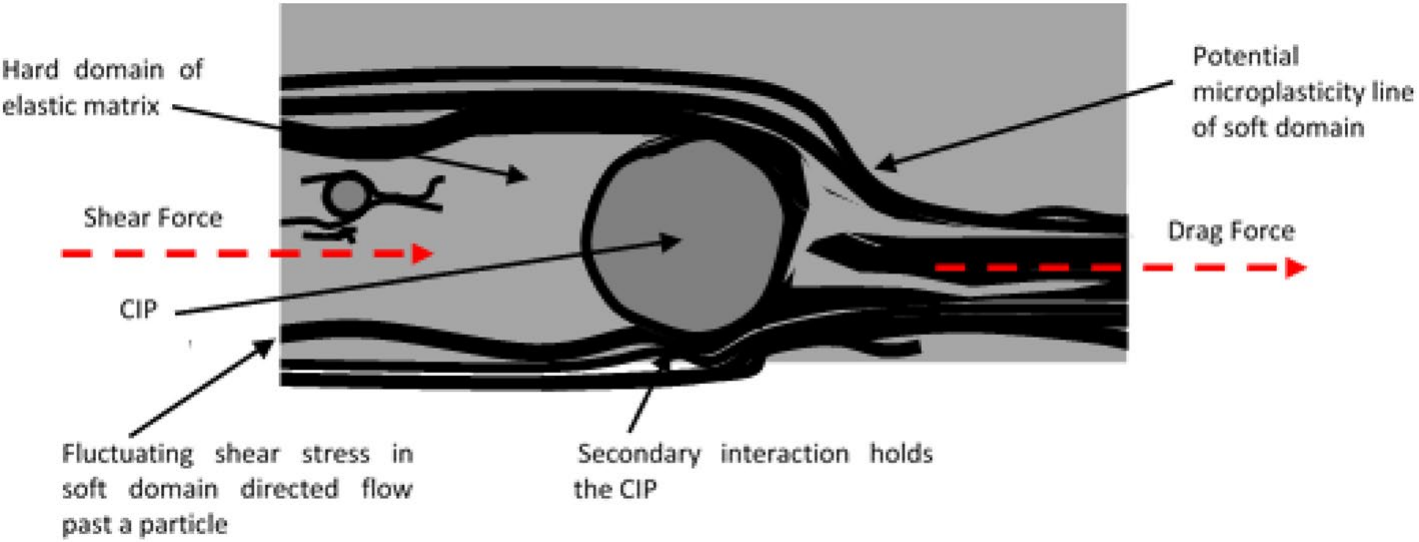
Schematic illustration of potential localized shear formation laterally past a particle.

In this investigation, the shear stress distributed uniformly to the region with less fluctuation in the primarily sheared region. As the fluctuated stress region was forced to remain localized in the shear band, a permanent microplasticity was developed. However, passing an obstructive particle, the soft domain of the shear band was forced to deviate alongside the hard domain molecular structure of the particle–matrix bond (i.e., secondary interaction). Besides, the dragged shear force after the deviation induced the microphase separation of shear bands, particularly between hard and soft domains. The shear force applied to the sample and passed through a particle at a localized soft domain is schematized in Fig. 11.

**Figure 12:**
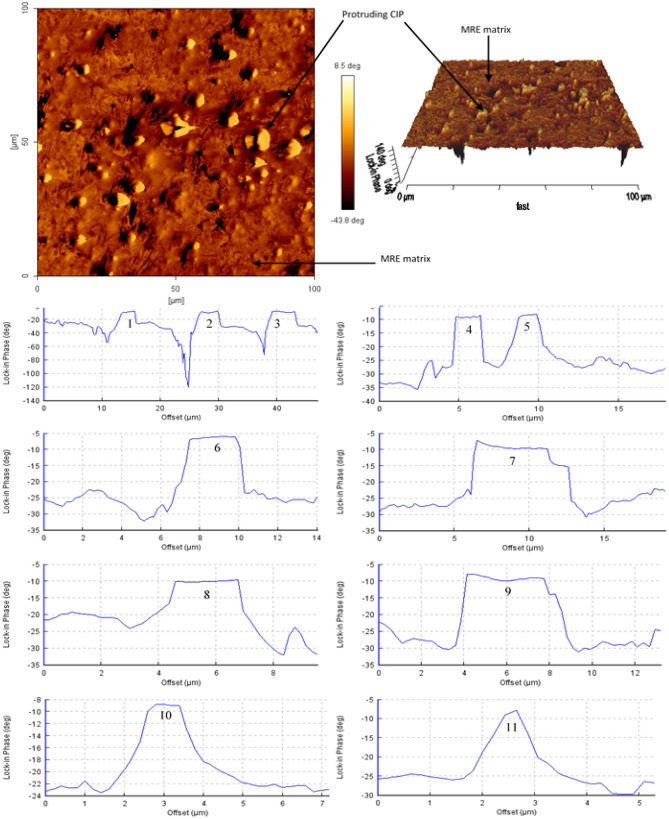
In-phase AFM image at localized strained region for particle measurement and 3D-AFM image processing of the sheared surface.

Determination of micro-size particles distribution and formation of micro-sized of microplasticity shear bands was analyzed using AFM, which is an important technique for characterization of microphase separation of the shear bands^20^. In this study, the sheared MRE sample was analyzed using the AFM tapping mode technique to identify surface topography and reliable identification of the differences between the region with different phase variations. In-phase image over 100 µm scan area obtained by this taping method is shown in Fig. 12. AFM 3D-image processing has validated previous morphology investigation where particles are more protruded in the localized strained surface of sheared sample with shear bands deformation.

**Figure 13:**
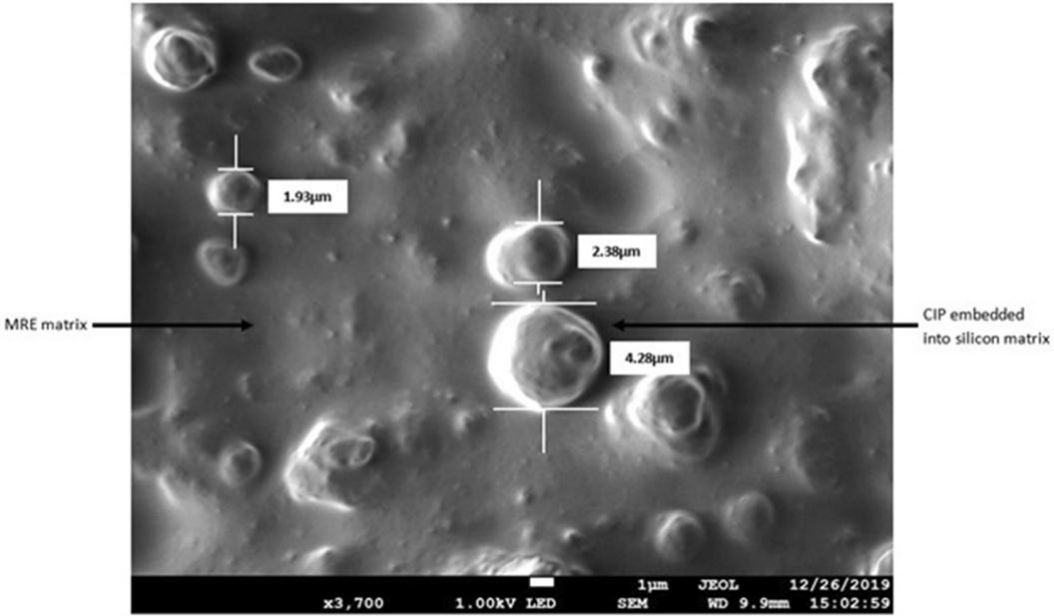
FESEM image of CIP in the matrix at various sizes.

Measurement of the average diameters of the homogenously distributed particles was achieved from this scanned region. The topography measurement indicated points of 1 to 11 representing the position of pultruded CIP been measured. At more than 10 different identified pultruded CIP, it was found that the particle diameter values are considered in a good agreement with the FESEM observation in Fig. 13 and that they also comply with the standard dimension given by the manufacturer.

**Figure 14:**
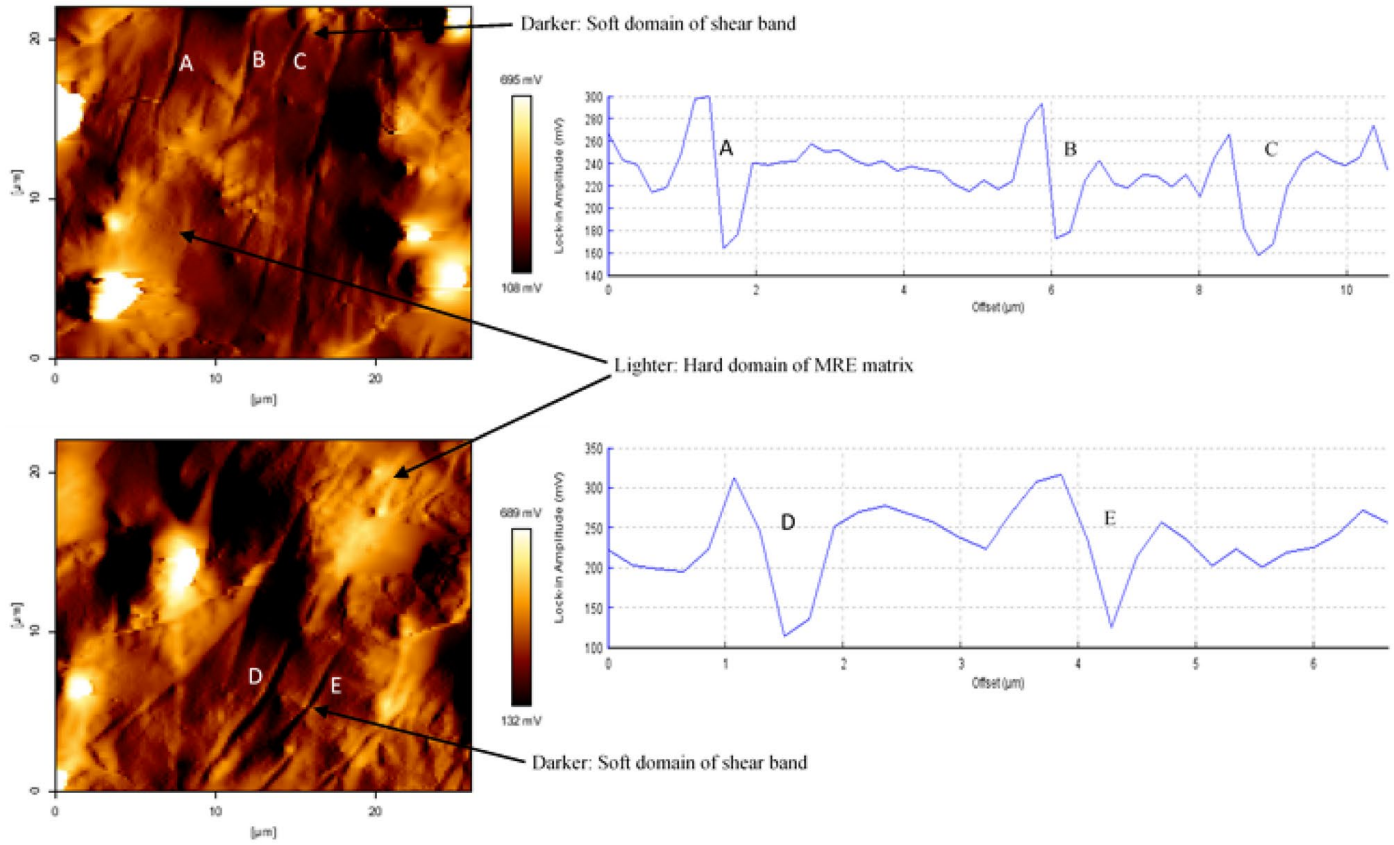
AFM tapping mode image and topography of MRE localized shear bands.

Morphological features of inter-domains and microphase separation of soft and hard localized domain regions can also be observed using this AFM taping method. This method systematically sets the phase offset of the harder domain with a lighter-color appearance whereas the softer domain appears darker^20,39^. These offsets concomitantly represent the modulus of the individual domain within a multi-domain of MRE resultant from stress relaxation durability shear test. Figure 14 clearly shows that a higher modulus domain of the matrix shown lighter color whereas, the lower modulus domain of shear bands appears darker. These observations agree with the expectation that shear bands were an amalgamation of micro-plastically stretched amorphous molecular chain structure. As the molecular chain stretched beyond the elastic limits of the localized strain region, cross-linkages suffered from rupturing, and without (little) cross-linkages, the chain uneasily reconfigured resulting in permanent transformation of plasticity. Micro-sized plasticity localized domain developed from this procedure originated shear bands ‘scar’ with softer domain as indicated by the darker color appearance in the AFM image.

A localized scan area of approximately 20 µm has permitted an advanced evaluation of the shear bands. Utilizing the topography measuring function of AFM analysis, the shear band of *A* is 0.53 µm in width, *B* is 0.66 µm, *C* is 0.97 µm, *D* is 0.85 µm and *E* is 0.83 µm. The depth of the shear bands concomitantly elucidated from the surface topographies chart. Considering these enumerated values corresponding to the confirmation of domain homogeneity, it can be presumed that the evaluated area using the AFM method was immense enough to represent the average results. The achieved data at least reasonably reflects the expectation of demarcating characteristics within the soft and hard domain. Furthermore, it is adequate to conclude that these behaviors are indistinguishable through the localized sheared regions of the MRE sample.

## Methods

### Preparation of MRE

The isotropic MRE sample-based on silicone rubber (SR) was fabricated by incorporating 70 wt% of soft carbonyl iron particles (CIP) (d50 = 3.8–5.3 µm, CC grade, supplied by BASF, Germany) into room temperature vulcanized (RTV) silicone rubber at 25 °C. The decision to opt for a 70 wt% CIP was taken in line with the literature^43^ and previous work on similar materials. Silicone rubber and iron particles were mixed in a beaker then mechanically stirred at a speed of 200 rpm and the mixture (NS625tds) was subsequently mixed with 0.1 wt% cross-linking agent (Nippon Steel Co., Japan). The agent was added for the curing process and followed the procedure provided by the Nippon Steel technical bulletin^44^. The whole mixture was then slowly poured on the mold base and spread evenly to ensure good surface finishing of the cured MRE. The curing process was carried out for 2 h at room temperature in a cylindrical closed-mold with a 50 mm diameter and 1.2 mm thickness of the circular sinking section inside the mold at off-state condition (no external magnetic stimuli). The degassing protocol was followed to eliminate the presence of air bubbles in the sample. Excessive matrix bleed-out along the edges of the MRE disc sheet was removed using a trimming knife. Finally, MRE circular disc sample was cut out using 23 mm hollow hole punch tools for dynamic testing.

### Measurement of durability by stress relaxation

The shear stress relaxation behavior of the MRE samples were tested under torsional shear mode using an oscillation parallel plate rheometer (Physica MCR 302, Anton Paar Company, Graz, Austria). MRE circular samples with 23 mm diameter and 1.2 mm nominal thickness were used. The rheometer was priorly initialized to the desired test condition (temperature, force, gap) and the measuring tool was aligned and attached to the quick connector coupling. A rotary disc parallel plate (pp20 rod) with a 20 mm diameter and 1 mm thickness was used. The sample was centrally placed on the stationary base mount of the rheometer and preloaded before the oscillation using rotary disc and then subjected to an initial normal force of 5.9 N to avoid wall slip^45^. The study^46^ suggested a technique used to have low initial normal force, but this technique was not commonly used by other researchers to investigate shear deformation under oscillatory rheometric and no available literature in the stress relaxation study was used the technique in their analysis. The measurement of significant shear stress relaxation parameters was conducted using the oscillatory test method which the measuring device oscillated around the axis during the test. The shear deformation was set at 0.01% constantly throughout the test and this was highlighted as the pristine attempt to stress relaxation test for MRE samples nearest to its state of rest to known as an equilibrium condition. The value was obtained by linear viscoelastic (LVE) determined on the basis of the visual technique and the procedure used in the literature^47^. Earlier, the amplitude sweep data for the LVE measurement was acquired from the rheological test of similar materials. The test frequencies were set at 1 Hz all the time to ideally simulate the real working condition in the application and consideration of the influenced by the shear velocity gradient during the test. The time interval of each test condition was set at every 4000 s and the total duration of the test reached up to 84,000 s to allow a wider range of behavior observation.

**Figure 15:**
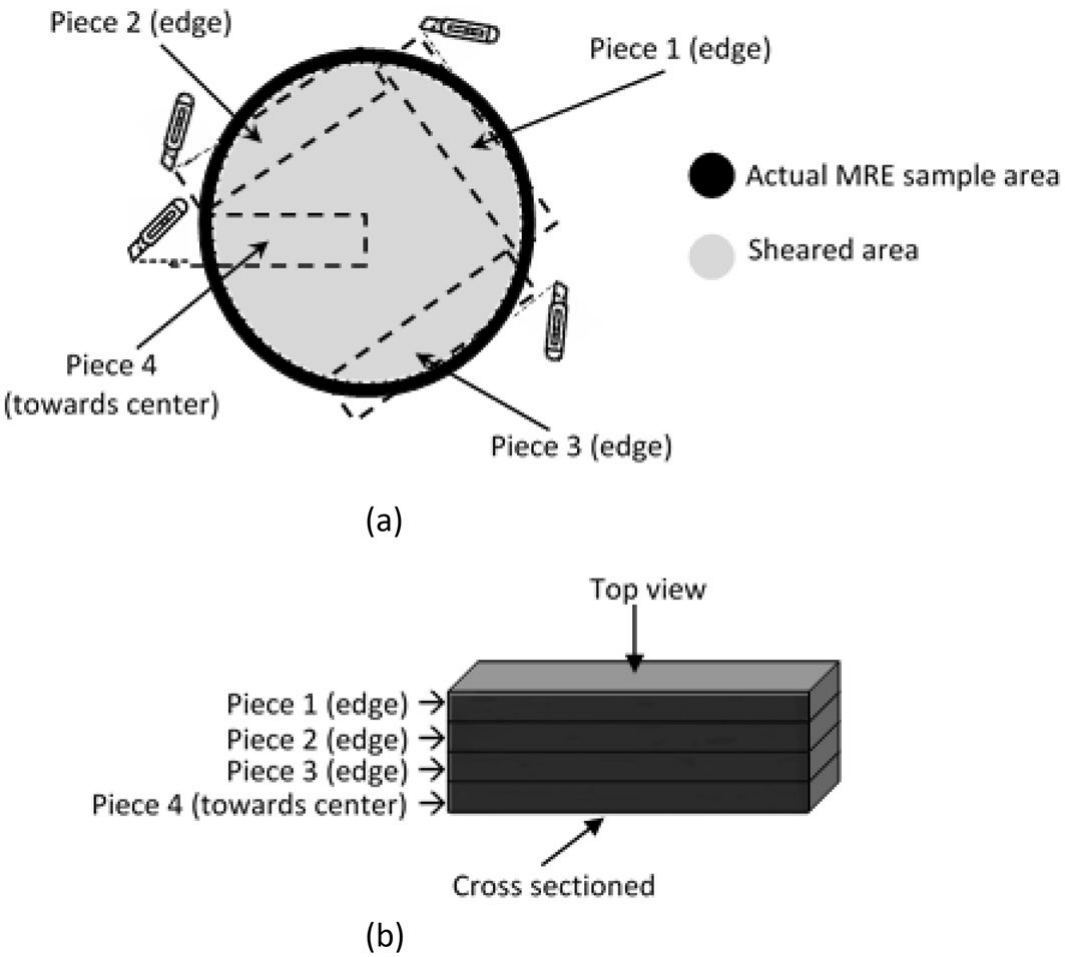
MRE sample (**a**) top view of the cutting location, and (**b**) set of cross-sectioned sheared edges and towards the sample’s center.

Samples for microstructure observation were prepared before and after the durability test by cutting them using utility knife with an extremely sharp blade steadily set perpendicular to the cutting edge of the sample (around the edges area of the MRE) into 1 mm X 10 mm. The cut area was selected based on the understanding that the shear center point of the sample was not caused by any torsional deformation (imaginary point), compared to the shearing acted on the edge of the sample which contributed to the maximum angular displacement. One of the pieces was cut from the edge towards the center of the sample to verify the midpoint condition as shown in Fig. 15a. A total of four trimmed pieces (from one sample) were then stacked to each other to get a complete evaluation of each edge’s cross-sectional area and the area closest to the center as shown in Fig. 15b. The correlation and feasible extent of data for each cross-sectioned are expected to represent the actual condition related to the randomly dispersed particles in the matrix. Both sample’s surfaces were sputter-coated with 1 nm thick platinum using an auto fine coater device (JEC-3000FC, JEOL, Japan) operated at 20 mA for 25 s. Subsequently, samples were placed into holders and investigated with a field emission scanning electron microscope (FESEM, JSM-7800F Prime, JOEL, Japan) operated an accelerating voltage of 1 kV. We have carried out a preliminary and potential analysis on the other related sample. We've tried a few places on the sample and we've seen them under FESEM. No proof of shear band is found other than the position chosen as shown in Fig. 15.

Observation for morphological studies on the MRE sample was further investigated using tapping-mode AFM analysis. This open-loop mode operated on a NanoWizard 3, NanoOptics AFM (JPK Instruments, Germany) using a nanosensor tapping-mode monolithic-silicon AFM probe-type single-beam cantilever supplied by BudgetSensors, USA. The cantilever had a nominal length of 125 µm and a nominal force constant 40 N/m, and a resonance frequency of 300 kHz. The non-coated tip of the cantilever having a rotated shape with a height of 17 µm and a radius of less than 10 nm. The initial scan area Details dimension design and full technical data of the AFM probes provided by manufacturer Budget Sensors (Innovative Solutions Bulgaria Ltd., Bulgaria).was set at 100 µm and details evaluation of the sheared sample with a particular failure mechanism was settled at approximately 20 µm scan area. Packaged analysis software was manipulated for the measurement and phase images.

## Conclusions

Elastomer based magnetorheological material consisting of silicone rubber matrix was successfully fabricated with 70 wt% CIP and cured under natural conditions without an external magnetic field. Morphological evaluation by FESEM images revealed that CIP was uniformly disseminated and well bonded in the silicon matrix. The durability performance of MRE was comprehensively investigated by the stress relaxation assessment at a constant 0.01% strain within the LVE region for a total of 84,000 s test duration. At such low constant strain, and a relatively long test period, this study provides important contribution to understanding the MRE system towards stress relaxation behavior. In consequence, stress relaxation performance was reduced by 6.9% throughout the test period. Rheological evaluation by storage modulus subjected to stress relaxation was found to decrease by 8.7%. The accompanying shear-deformation durability that related to the normal force over the same test period was also investigated and found to gradually decrease by 27%. Concession to the MRE performance at storage modulus, normal force, and stress relaxation have motivated the extensive analysis of the possible phenomena at the microscopic level. The cross-sectioned surface condition of sheared samples was investigated via FESEM and exhibited a new finding of the failure mechanism in MRE. Stress relaxation has developed strain localization and produced microplasticity in very confined regions called shear bands. The amorphous molecular chain structure and cross-linkages of the matrix were suggested to be responsible for this permanent plasticity mechanism. Morphological analysis and measurement of the appeared shear bands and related deformations that have never been implemented in the investigation of MRE stress relaxation were then evaluated by AFM for the first time. Physical features of the shear bands have been successfully measured using the AFM topography measuring technique. Thus, the material performance and failure become a critical aspect, particularly the elastic failure mechanism. Additionally, different orientation may produce less shear band formation compared to current study (isotropic) and may take longer duration to create the shear band. This may, however, remain as a hypothesis until scientific research has been carried out. Overall, the results presented in this study successfully manifest the variation of possible failure mechanisms for stress relaxation durability in MRE and provide a critical assessment for the development of material quality and performance.

## References

[1] Li, W. H., Zhang, X. Z. & Du, H. Magnetorheological Elastomers and Their Applications. in *Advances in Elastomers I: Blends and Interpenetrating Networks* (eds. Visakh, P. M., Thomas, S., Chandra, A. K. & Mathew, A. P.) 357–374 (Springer Berlin Heidelberg, 2013). 10.1007/978-3-642-20925-3_12.

[2] Qiao X (2012). Microstructure and magnetorheological properties of the thermoplastic magnetorheological elastomer composites containing modified carbonyl iron particles and poly(styrene-b-ethylene-ethylenepropylene-b-styrene) matrix. Smart Mater. Struct..

[3] Li WH, Nakano M (2013). Fabrication and characterization of PDMS based magnetorheological elastomers. Smart Mater. Struct..

[4] Xu, L., Zou, A., Fu, J., Yu, M. & Bai, J. Development and simulation evaluation of a magnetorheological elastomer isolator for transformer vibration control. in *2018 Chinese Control And Decision Conference (CCDC)* vol. 23 2600–2604 (IEEE, 2018).

[5] Prabhakar R. Marur. U.S Patent 20130087985A1. (2013).

[6] Rodenbeck, P. D. U.S Patent 008176958B2. (2012).

[7] Ubaidillah SJ, Purwanto A, Mazlan SA (2015). Recent progress on magnetorheological solids: materials, fabrication, testing, and applications. Adv. Eng. Mater..

[8] Stevenson, A. & Campion, R. Durability. in *Engineering with Rubber* vol. 3rd editio 205–257 (Carl Hanser Verlag GmbH &amp; Co. KG, 2012).

[9] Faizal Johari MA (2020). An overview of durability evaluations of elastomer-based magnetorheological materials. IEEE Access.

[10] Wang Y, Gong X, Yang J, Xuan S (2014). Improving the dynamic properties of MRE under cyclic loading by incorporating silicon carbide nanoparticles. Ind. Eng. Chem. Res..

[11] Gorman D, Murphy N, Ekins R, Jerrams S (2016). The evaluation and implementation of magnetic fields for large strain uniaxial and biaxial cyclic testing of magnetorheological elastomers. Polym. Test..

[12] Bastola AK, Paudel M, Li L, Li W (2020). Recent progress of magnetorheological elastomers: a review. Smart Mater. Struct..

[13] Karrabi M, Mohammadian-Gezaz S (2011). The effects of carbon black-based interactions on the linear and non-linear viscoelasticity of uncured and cured SBR compounds. Iran. Polym. J..

[14] Yu K, Ge Q, Qi HJ (2014). Reduced time as a unified parameter determining fixity and free recovery of shape memory polymers. Nat. Commun..

[15] Lu H, Huang WM (2013). On the origin of the Vogel–Fulcher–Tammann law in the thermo-responsive shape memory effect of amorphous polymers. Smart Mater. Struct..

[16] Lu H, Du S (2014). A phenomenological thermodynamic model for the chemo-responsive shape memory effect in polymers based on Flory-Huggins solution theory. Polym. Chem..

[17] Johari MAF (2021). Shear band formation in magnetorheological elastomer under stress relaxation. Smart Mater. Struct..

[18] Tobolsky AV (1956). Stress relaxation studies of the viscoelastic properties of polymers. J. Appl. Phys..

[19] Abu-Abdeen M (2010). Single and double-step stress relaxation and constitutive modeling of viscoelastic behavior of swelled and un-swelled natural rubber loaded with carbon black. Mater. Des..

[20] Xia H, Song M, Zhang Z, Richardson M (2007). Microphase separation, stress relaxation, and creep behavior of polyurethane nanocomposites. J. Appl. Polym. Sci..

[21] Liu X, Dong X, Liu W, Han CC, Wang D (2018). Morphology evolution and dynamic relaxation behavior of solution-polymerized styrene-butadiene rubber/polyisoprene/silica ternary composites influenced by shear. Polymer (Guildf)..

[22] Schmitt JA, Keskkula H (1960). Short-time stress relaxation and toughness of rubber-modified polystyrene. J. Appl. Polym. Sci..

[23] Jagla EA (2007). Strain localization driven by structural relaxation in sheared amorphous solids. Phys. Rev. E.

[24] Qi S, Yu M, Fu J, Zhu M (2018). Stress relaxation behavior of magnetorheological elastomer: experimental and modeling study. J. Intell. Mater. Syst. Struct..

[25] Yu GJ, Lin XG, Guo F (2017). Modeling and verification of relaxation behavior for magnetorheological elastomers with applied magnetic field. Key Eng. Mater..

[26] Lai NT, Ismail H, Abdullah MK, Shuib RK (2019). Optimization of pre-structuring parameters in fabrication of magnetorheological elastomer. Arch. Civ. Mech. Eng..

[27] Boczkowska A, Awietjan SF, Wroblewski R (2007). Microstructure–property relationships of urethane magnetorheological elastomers. Smart Mater. Struct..

[28] Gong XL, Chen L, Li JF (2007). Study of utilizable magnetorheological elastomers. Int. J. Mod. Phys. B.

[29] Tian TF, Li WH, Alici G, Du H, Deng YM (2011). Microstructure and magnetorheology of graphite-based MR elastomers. Rheol. Acta.

[30] Ubaidillah (2019). Swelling, thermal, and shear properties of a waste tire rubber based magnetorheological elastomer. Front. Mater..

[31] Boczkowska A, Awietjan SF, Wejrzanowski T, Kurzydłowski KJ (2009). Image analysis of the microstructure of magnetorheological elastomers. J. Mater. Sci..

[32] Liao G, Gong X, Xuan S (2013). Influence of shear deformation on the normal force of magnetorheological elastomer. Mater. Lett..

[33] Menzel AM (2019). Mesoscopic characterization of magnetoelastic hybrid materials: magnetic gels and elastomers, their particle-scale description, and scale-bridging links. Arch. Appl. Mech..

[34] Han, Y., Zhang, Z., Faidley, L. E. & Hong, W. Microstructure-based modeling of magneto-rheological elastomers. in *Proceeding of SPIE-Behavior and Mechanics of Multifunctional Materials and Composites 2012* (eds. Goulbourne, N. C. & Ounaies, Z.) vol. 8342 83421B (2012).

[35] Gent AN (1962). Relaxation processes in vulcanized rubber. II. Secondary relaxation due to network breakdown. J. Appl. Polym. Sci..

[36] Maria HJ (2014). Stress relaxation behavior of organically modified montmorillonite filled natural rubber/nitrile rubber nanocomposites. Appl. Clay Sci..

[37] Valiev KK, Minaev AY, Stepanov GV, Karnet YN, Yumashev OB (2019). Scanning Probe Microscopy of Magnetorheological Elastomers. J. Surf Investig. X-ray, Synchrotron Neutron Tech..

[38] Valiev HH (2016). Atomic force microscopy and physical-Mechanical properties of new elastomer composites. Mater. Phys. Mech..

[39] Iacobescu GE, Balasoiu M, Bica I (2013). Investigation of surface properties of magnetorheological elastomers by atomic force microscopy. J. Supercond. Nov. Magn..

[40] Fuchs A, Sutrisno J, Gordaninejad F, Caglar MB, Yanming L (2010). Surface polymerization of iron particles for magnetorheological elastomers. J. Appl. Polym. Sci..

[41] Budrikis Z, Castellanos DF, Sandfeld S, Zaiser M, Zapperi S (2017). Universal features of amorphous plasticity. Nat. Commun..

[42] Cullity BD (1978). Elements of X-Ray Diffraction.

[43] Davis LC (1999). Model of magnetorheological elastomers. J. Appl. Phys..

[44] Nippon Steel. *Nippon Steel Technical Bulletin NS 625 A & B*. (2000).

[45] Shabdin M (2019). Material characterizations of Gr-based magnetorheological elastomer for possible sensor applications: rheological and resistivity properties. Materials (Basel)..

[46] Walter BL, Pelteret J-P, Kaschta J, Schubert DW, Steinmann P (2017). Preparation of magnetorheological elastomers and their slip-free characterization by means of parallel-plate rotational rheometry. Smart Mater. Struct..

[47] Agirre-Olabide I, Berasategui J, Elejabarrieta MJ, Bou-Ali MM (2014). Characterization of the linear viscoelastic region of magnetorheological elastomers. J. Intell. Mater. Syst. Struct..

